# Intrafraction tumor motion monitoring and dose reconstruction for liver pencil beam scanning proton therapy

**DOI:** 10.3389/fonc.2023.1112481

**Published:** 2023-03-02

**Authors:** Saber Nankali, Esben Schjødt Worm, Jakob Borup Thomsen, Line Bjerregaard Stick, Jenny Bertholet, Morten Høyer, Britta Weber, Hanna Rahbek Mortensen, Per Rugaard Poulsen

**Affiliations:** ^1^ Danish Centre for Particle Therapy, Aarhus University Hospital, Aarhus, Denmark; ^2^ Department of Clinical Medicine, Aarhus University, Aarhus, Denmark; ^3^ Department of Oncology, Aarhus University Hospital, Aarhus, Denmark; ^4^ Division of Medical Radiation Physics and Department of Radiation Oncology, Inselspital, Bern University Hospital, and University of Bern, Bern, Switzerland

**Keywords:** proton therapy, pencil beam scanning, dose reconstruction, liver cancer, motion management, respiratory gating, tumor motion monitoring (Min.5-Max. 8)

## Abstract

**Background:**

Pencil beam scanning (PBS) proton therapy can provide highly conformal target dose distributions and healthy tissue sparing. However, proton therapy of hepatocellular carcinoma (HCC) is prone to dosimetrical uncertainties induced by respiratory motion. This study aims to develop intra-treatment tumor motion monitoring during respiratory gated proton therapy and combine it with motion-including dose reconstruction to estimate the delivered tumor doses for individual HCC treatment fractions.

**Methods:**

Three HCC-patients were planned to receive 58 GyRBE (n=2) or 67.5 GyRBE (n=1) of exhale respiratory gated PBS proton therapy in 15 fractions. The treatment planning was based on the exhale phase of a 4-dimensional CT scan. Daily setup was based on cone-beam CT (CBCT) imaging of three implanted fiducial markers. An external marker block (RPM) on the patient’s abdomen was used for exhale gating in free breathing. This study was based on 5 fractions (patient 1), 1 fraction (patient 2) and 6 fractions (patient 3) where a post-treatment control CBCT was available. After treatment, segmented 2D marker positions in the post-treatment CBCT projections provided the estimated 3D motion trajectory during the CBCT by a probability-based method. An external-internal correlation model (ECM) that estimated the tumor motion from the RPM motion was built from the synchronized RPM signal and marker motion in the CBCT. The ECM was then used to estimate intra-treatment tumor motion. Finally, the motion-including CTV dose was estimated using a dose reconstruction method that emulates tumor motion in beam’s eye view as lateral spot shifts and in-depth motion as changes in the proton beam energy. The CTV homogeneity index (HI) The CTV homogeneity index (HI) was calculated as $\frac{\text{D}2\text{\% }-\text{ D}98\%}{\text{D}50\%}\;\times 100\%$ .

**Results:**

The tumor position during spot delivery had a root-mean-square error of 1.3 mm in left-right, 2.8 mm in cranio-caudal and 1.7 mm in anterior-posterior directions compared to the planned position. On average, the CTV HI was larger than planned by 3.7%-points (range: 1.0-6.6%-points) for individual fractions and by 0.7%-points (range: 0.3-1.1%-points) for the average dose of 5 or 6 fractions.

**Conclusions:**

A method to estimate internal tumor motion and reconstruct the motion-including fraction dose for PBS proton therapy of HCC was developed and demonstrated successfully clinically.

## 1 Introduction

Radiation therapy is a local treatment option for small hepatocellular carcinoma (HCC) tumors in inoperable patients with a good liver function (Child-Pugh A) (1). However, HCC patients often present with a considerable tumor burden and an underlying cirrhotic liver and even low doses of radiation to the liver leads to a high risk of developing radiation induced liver disease (RILD) in patients with cirrhosis (2). Since RILD is a severe condition that can lead to liver failure and death, it is crucial to minimize the dose to the normal liver tissue surrounding the tumor (3).

Compared to photon based radiation therapy, pencil beam scanning (PBS) proton therapy can often provide more conformal target dose distributions with less healthy tissue irradiation (4, 5). Proton therapy is therefore increasingly used in the treatment of HCC (6, 7). However, liver tumors often exhibit large and variable respiratory motion during treatment (8), which can cause considerable deviations between the delivered and planned doses. Due to interplay effects and a high sensitivity to water equivalent path length changes, PBS proton therapy is particularly prone to dosimetric uncertainties caused by target motion (9–11) and international guidelines underline the special need for motion management in PBS proton therapy (12, 13). Hence, respiratory gating, where the beam is only turned on during specific phases of the breathing cycle has been proposed and implemented in proton therapy to mitigate tumor motion effects (14–17). Still, residual motion within the gating window is of concern (16, 18).

Reconstruction of the actual delivered tumor dose at a fraction requires knowledge of the internal motion during treatment delivery and synchronization of this motion with the beam delivery. One method is to calculate the dynamic 4D dose (D4DD) by ascribing a specific phase of a 4DCT scan to each delivered spot, use this to calculate phase-specific doses in each 4DCT phase and accumulate these doses in a reference phase by deformable image registration (12, 13). This method has been implemented clinically for PBS carbon therapy (19) and proton therapy (20, 21) using a waist belt for respiratory monitoring during beam delivery. A limitation of the D4DD is that it neglects setup errors and assumes that the internal motion during treatment is well described by the respiration signal and identical to the motion in the 4DCT. However, liver motion is known to be highly variable and often poorly represented by 4DCT scans that by nature only capture one (random) respiratory cycle at each longitudinal position within the patient (22, 23). To overcome these limitations, Yamada et al. monitored the internal motion of implanted fiducial markers in the liver during gated proton PBS delivery by a gantry-mounted stereoscopic fluoroscopic x-ray imager (24). By combining the internal motion with the spot delivery timing in machine log files the authors reconstructed the tumor dose by a spot shift method that can account for arbitrary rigid motion (25). However, although many modern conventional proton facilities are equipped with dual x-ray imagers, these can typically not be used during treatment delivery. Consequently, target motion monitoring during treatment is normally not available even though it is recommended by international guidelines for proton therapy of moving targets (12, 13).

In this study, we introduce a method to overcome the limitations of conventional proton facilities in internal tumor motion monitoring during proton PBS treatment. The method uses an external-internal correlation model (ECM) to estimate the internal tumor motion from an external respiratory signal and combines the internal motion with spot delivery timing in machine log files to estimate the tumor position during each spot delivery. The motion is then combined with the spot shift dose reconstruction method to estimate the tumor doses for individual HCC treatment fractions.

## 2 Material and methods

### 2.1 Patients and treatment planning

Three patients with HCC underwent proton PBS in April-September 2022 in a national phase II clinical trial that allowed inclusion of both large tumors and Child-Pugh B patients. The trial was approved by the relevant ethics committees (ClinicalTrials.gov Identifier: NCT05203120). Three gold or platinum fiducial markers with dimensions of 0.75 mm × 5 mm (Visicoil™) were implanted near the tumor the day before planning CT scanning. An internal clinical target volume (iCTV) was formed as the union of the CTV in the five phases of a 10-phase 4-dimensional CT scan (4DCT) that were closest to full exhale. It corresponded to exhale respiratory gating with approximately 50% duty cycle. A 3-field proton plan was created on the exhale phase of the 4DCT using a commercial treatment planning system (TPS, Eclipse 16.01.10, Varian, a Siemens Healthineers Company, Palo Alto, CA) and dose calculation algorithm (Varian Proton Convolution Superposition 16.1.0). Robust single field uniform dose (SFUD) optimization was performed with ±4.5% range uncertainty and ±5 mm shifts in the left-right (LR) and anterior-posterior (AP) directions and ±7 mm shifts in the cranio-caudal (CC) direction. The treatment plans used beam energies in the range 71-153 MeV, for which the spot size in air is 4-6mm (1 standard deviation). Each field had 528-2134 spots and a total of 6617-16047 monitor units. The prescribed mean iCTV dose was 58 GyRBE (Patient 1 and 3) for central tumors (≤2 cm from porta hepatis) or 67.5 GyRBE (Patient 2) for peripheral tumors (>2 cm from porta hepatis) in 15 fractions.

### 2.2 Treatment delivery and imaging

Daily patient setup was based on a free-breathing CBCT scan in which the estimated exhale positions of the motion-blurred fiducial markers were matched with the planning CT. CBCT scan was done using Standard ProBeam CBCT imaging system with Paxscan 4030CB flat panel detectors. The resolution of the image detector was 0.388 mm/pixel in both directions with source-to-imager distance (SID) of 3700 mm and source-to-axis distance (SAD) of 2700 mm. During the CBCT acquisition and throughout the whole treatment session the position of a marker block (Real-time Position Management System, RPM, Varian) on the patient’s abdomen was recorded with a camera. During treatment the RPM signal was used for respiratory gating with a gating window adjusted before treatment to obtain a duty cycle of approximately 50% centered around the exhale phase in accordance with the iCTV construction. A post-treatment control CBCT scan was captured at 6, 1, and 7 fractions for patients 1, 2, and 3, respectively. The RPM log file was missing for one of these treatment fractions for patient 1 and patient 3. The analysis presented in this study requires a post-treatment CBCT and an RPM log file and was therefore only made for 5, 1 and 6 fractions for patients 1, 2, and 3, respectively.

### 2.3 Data analysis

After the treatments the fiducial markers were segmented in each raw 2D CBCT-projection (~1000 images per CBCT) using an automatic method (26) followed by manual inspection and semi-automatic correction of failed segmentations. The 3D motion trajectory of each marker during CBCT was estimated by a probability-based method (27) and the marker group centroid motion was used as a surrogate for the tumor motion. The exhale period was defined as the time within the 95^th^-100^th^ percentile of the markers position in the CC direction for each CBCT. This was used to determine the exhale position in each direction of motion as the mean marker position during the exhale period. For the setup CBCT scans, the resulting exhale tumor position was used to determine the optimal setup couch correction for marker alignment with the planned marker positions in exhale. This is similar to the trajectory-based setup introduced for non-gated treatments in (28). The online registration error was then calculated as the difference between the retrospective trajectory-based patient setup and the actual couch correction based on online 3D/3D registration of the setup CBCT with the planning CT scan. Furthermore, the intrafraction baseline drift of the exhale position between the setup CBCT and the post-treatment CBCT was determined as the difference between their respective exhale positions.

**Figure 1:**
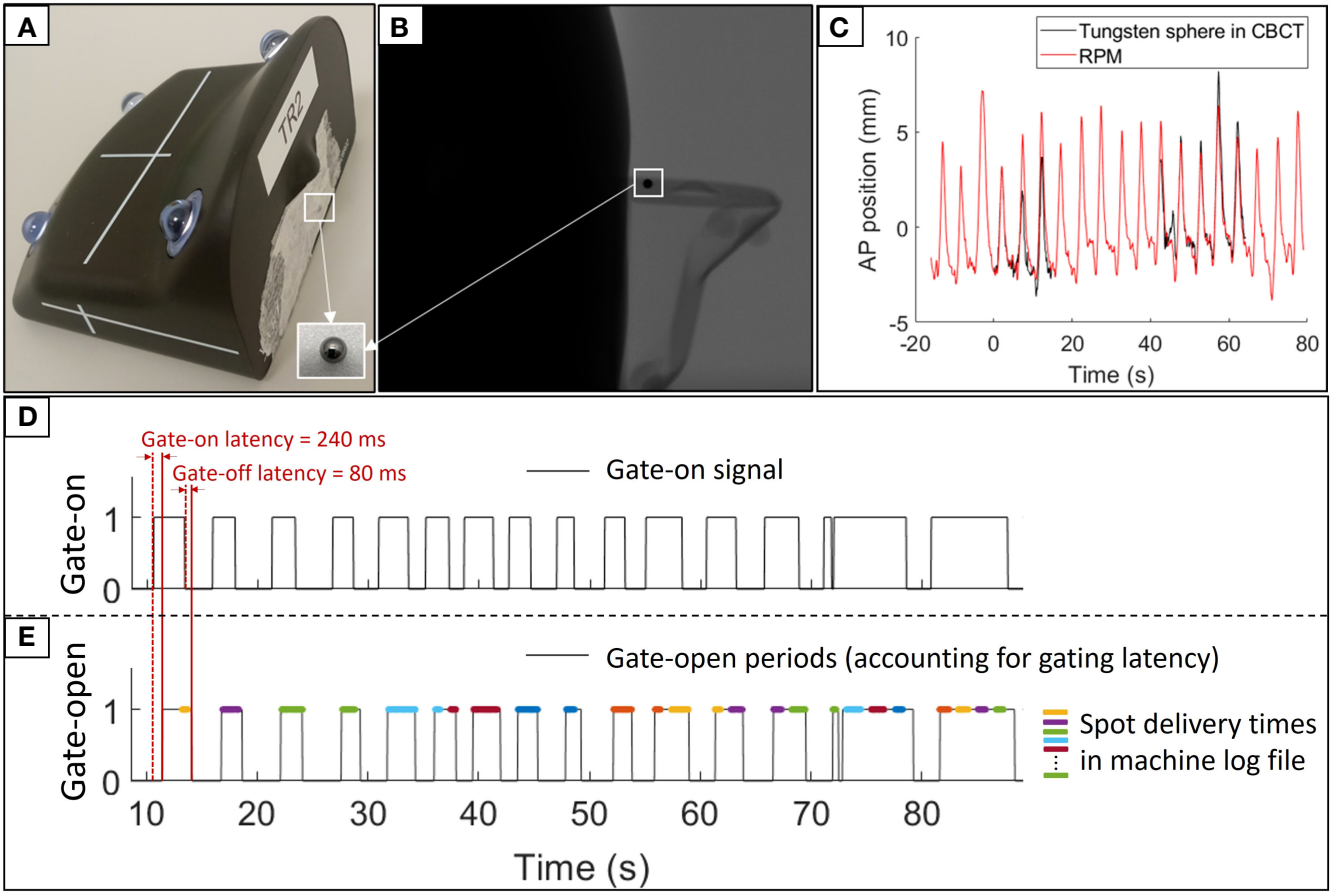
Synchronization of RPM log files. **(A)** Marker block with the tungsten sphere used for synchronization with the CBCT projections. **(B)** A CBCT projection showing the marker block and tungsten sphere. **(C)** Synchronized RPM signal (red) and anterior-posterior (AP) tungsten sphere trajectory extracted from the CBCT projections (black). **(D)** Gate-on signal in RPM log file used for synchronization with spot delivery times. **(E)** Gate-open times accounting for the gating latency (black) and synchronized spot delivery times from machine log files (colored lines, with different colors indicating different energy layers).

The analysis in this study required synchronization of the RPM signal with the projection images of the post-treatment CBCT (to establish an ECM) and with the delivery time of each proton spot (to perform dose reconstruction). Synchronization between RPM and CBCT projections was obtained by placing a 3 mm diameter tungsten sphere on the RPM block such that it was visible in most of the CBCT projections (**Figures 1A, B**). After treatment the 3D motion of the tungsten sphere during the CBCT scan was estimated from its projected motion in the CBCT projections (27), and its AP motion was temporally aligned with the RPM motion in gating log files (**Figure 1C**). This synchronization provided the logged RPM position at the acquisition time of each CBCT projection.

The synchronization between RPM and spot delivery times was based on the gate-on signal in the gating log files. The logged gate-on signal specifies the time intervals when the RPM block is inside the gating window, but it does not account for the gate-on latency between entering the gating window and beam-on and the gate-off latency between exiting the gating window and beam-off. The gate-open times during which the beam could potentially be turned on were estimated from the logged gate-on signal by assuming a gate-on latency of 240 ms and a gate-off latency of 80 ms (**Figure 1D**). These latencies were measured using the method proposed by Worm et al. (29) and rounded to an integer number of gating log file samples (40ms resolution). Next, a comparison of the gate-open times with the actual spot delivery times in machine log files (30) provided the synchronization between RPM log files and spot delivery times (**Figure 1E**). While the machine log files specified the duration of each spot with microsecond resolution it did not directly specify the beam-off times occurring during larger spots shifts, energy shifts and gate-off periods. However, by using the logged number of User Datagram Protocol (UDP) messages received between each spot the beam-off times were estimated with a scaling factor uncertainty of a few percent. During the synchronization of the machine log files with the gating log files the beam-off times were scaled to fit the time scale in the gating log file.

**Figure 2:**
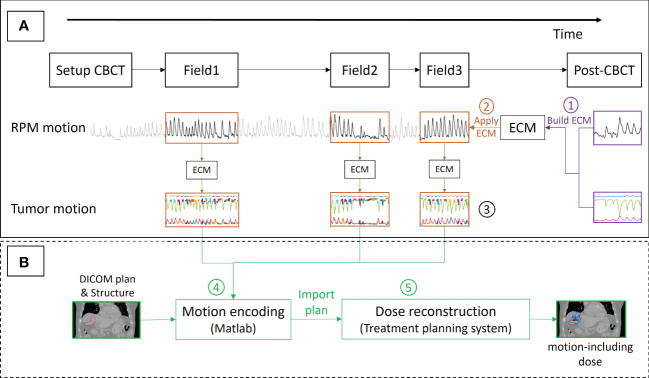
Workflow for **(A)** estimating the tumor position at the time of delivery of each spot by an external-internal correlation model (ECM) and **(B)** motion-including dose reconstruction. The numbers refer to the description in the text. The thick colored curves shown on the top of the tumor motion (Label 3) show the spot delivery times with different energy layers indicated with different colors.


To estimate the tumor motion during treatment delivery an augmented linear ECM that estimated the tumor motion during the post-treatment CBCT from the synchronized RPM motion (31) was built (Label 1 in **Figure 2**):

(1) INT(t) = A.EXT(t) + B.EXT(t-τ) + C

Here, INT and EXT are internal tumor and external RPM motion as a function of time (t). The coefficients A, B and C and the time delay τ are fitting parameters. The augmentation term B.EXT(t-τ) accounts for hysteresis and phase differences between internal and external motion (31). A, B and C were optimized individually for each motion direction with least-square fitting while the same value of τ was used for all three motion directions.

Next, The ECM was used to estimate the tumor motion throughout the treatment delivery from the intra-treatment RPM signal (Label 2). This synchronization resulted in the ECM estimated tumor position at the time of each spot delivery (Label 3). For each spot, the geometrical treatment accuracy was determined as the tumor position relative to the planned position. The tumor motion range was calculated for each fraction as the difference between the 98^th^ and the 2^nd^ percentiles of the tumor position over all three fields during beam-on periods and, for comparison, over the full field durations including both beam-on and beam-off periods.

Finally, the CTV dose and the dose to the healthy liver tissue of each analyzed fraction was estimated by a motion-including dose reconstruction method that emulates tumor motion in beam’s eye view by shifting each spot in the opposite direction of the tumor motion and in-depth tumor motion as changes in the proton beam energy (25). A motion-encoded plan with these manipulations of the original spot positions and energies was created by an in-house developed Matlab program (Label 4) and imported into the TPS for recalculation (Label 5). The reconstructed CTV doses of each fraction and averaged over all analyzed fractions were compared with the planned doses using the metrics of CTV D98% and D2% (minimum dose received by 98% and 2% of the CTV volume) and the homogeneity index $\text{HI\%}=\frac{\text{D}2\text{\% }-\text{ D}98\%}{\text{D}50\%}\;\times 100\%$ . Furthermore, the mean healthy liver tissue doses averaged over all analyzed fractions were compared with the planned doses.

## 3 Results

**Figure 3:**
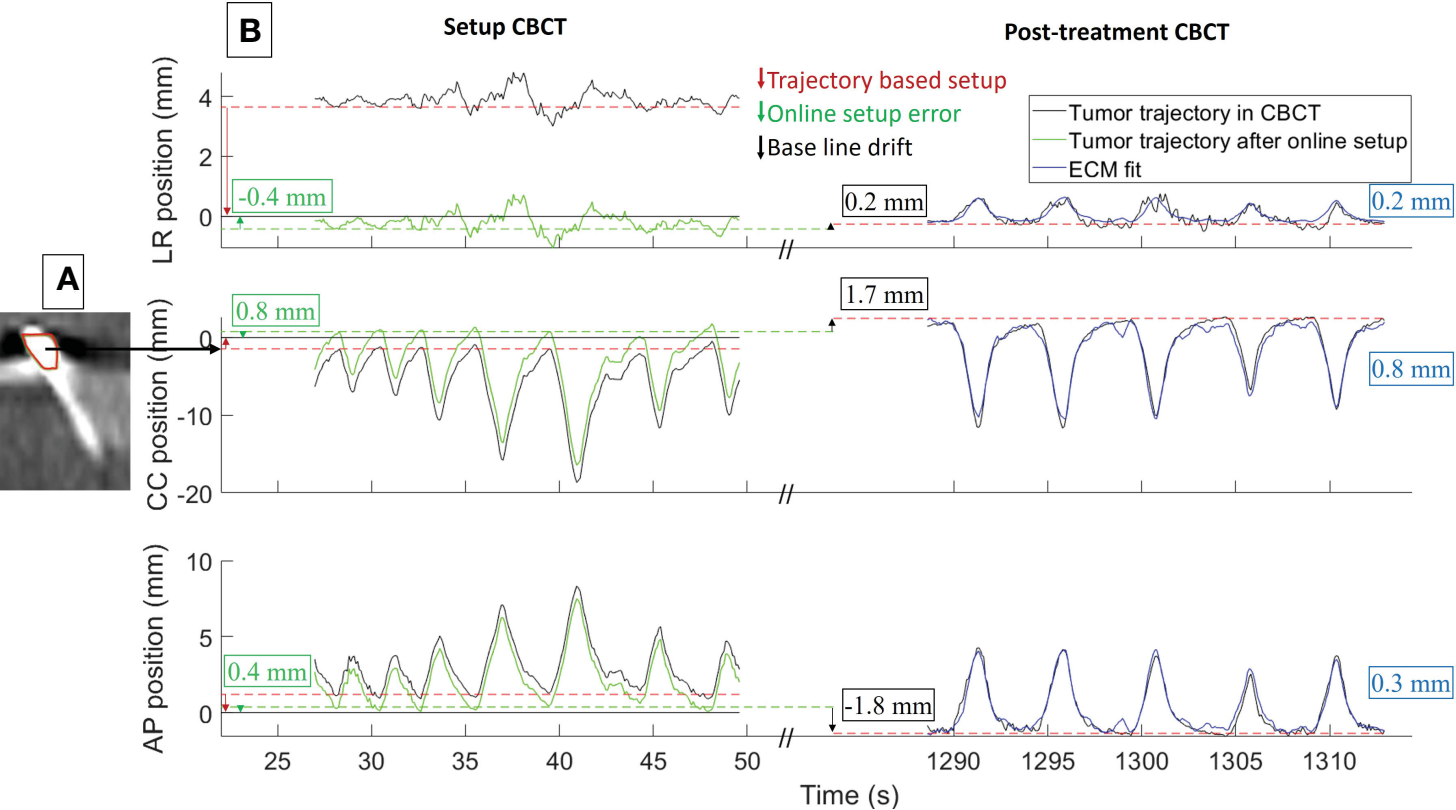
Example of **(A)** reconstructed CBCT and **(B)** tumor motion during CBCT (patient 3, fraction 3). **(A)** Blurred marker in the setup CBCT scan and the online registration to the planned exhale marker position (red contour). **(B)** Estimated 3D tumor trajectories during setup CBCT and post-treatment CBCT relative to the planned position (black curves), tumor trajectory in setup CBCT after online registration and couch correction (green curves), and external-internal correlation model (ECM) fit for the post-treatment CBCT (blue curves). The numbers show the online registration error (green), intrafraction baseline drift (black), and root mean square error of ECM fit (blue). LR, left-right; CC, cranio-caudal; AP, anterior-posterior.

**Figure 3** shows an example of the tumor motion trajectory during the setup CBCT and the post-treatment CBCT at one fraction for patient 3. At this fraction, the online registration errors were 0.4-0.8 mm (**Figure 3**). The mean online registration error was in general sub-millimeter for all patients (**Table 1**). The example case in **Figure 3** had a small cranial and posterior drift of the tumor exhale position from setup CBCT to post-treatment CBCT (black arrows). Averaged over all fractions and patients a similar trend was seen with mean ± standard deviation (SD) drift motion of 0.0 mm ± 0.8 mm (LR), 1.3 mm ± 1.3 mm (CC), and -0.7 mm ± 1.0 mm (AP) (**Table 1**).

**Table 1:** Mean ± standard deviation over all fractions of the online registration error, baseline drift, root-mean-square (RMS) ECM fitting error, tumor position error during spot delivery (treatment error), and the tumor motion range (2^nd^ to 98^th^ percentile motion) during beam-on and during field delivery regardless of beam-on status.

Patient	1			2			3		
Direction	LR (mm)	CC (mm)	AP (mm)	LR (mm)	CC (mm)	AP (mm)	LR (mm)	CC (mm)	AP (mm)
Online registration error	-0.7 ± 0.4	-0.3 ± 0.9	-0.4 ± 0.5	-0.9	-0.8	0.8	0.3 ± 0.6	1.0 ± 1.0	-0.2 ± 0.5
Baseline drift	-0.1 ± 0.5	1.4 ± 1.4	-0.8 ± 0.4	-1.2	2.0	1.7	0.3 ± 0.9	1.2 ± 1.3	-1.0 ± 0.8
RMS error of ECM fit	0.9 ± 0.3	2.1 ± 0.8	1.5 ± 0.6	0.7	1.4	0.7	0.2 ± 0.0	0.9 ± 0.2	0.5 ± 0.1
Treatment error during beam-on	-1.6 ± 1.1	-0.7 ± 3.0	0.2 ± 2.4	-1.7 ± 0.4	-0.6 ± 1.3	2.9 ± 0.5	0.1 ± 0.7	-0.7 ± 2.8	0.1 ± 1.2
Tumor motion range during beam-on periods	2.7 ± 0.3	10.9 ± 1.6	7.4 ± 1.0	1.8	5.0	2.1	0.4 ± 0.2	6.8 ± 1.3	3.5 ± 0.7
Full tumor motion range during beam-on and beam-off periods	5.1 ± 0.8	23.8 ± 2.7	15.9 ± 2.1	3.9	13.9	6.1	0.9 ± 0.5	15.9 ± 2.1	7.7 ± 1.1

LR, left-right; CC, cranio-caudal; AP, anterior-posterior.

The example ECM presented in **Figure 3** (blue curves) had an accuracy close to the mean RMS fit error for patient 3, while patients 1 and 2 had larger RMS fit errors up to 2.1 mm (**Table 1**). Over all patients, the mean ( ± SD) RMS fit error of the ECM was 0.5 mm ± 0.4 mm (LR), 1.5 mm ± 0.8 mm (CC), and 1.0 mm ± 0.6 mm (AP).

**Figure 4:**
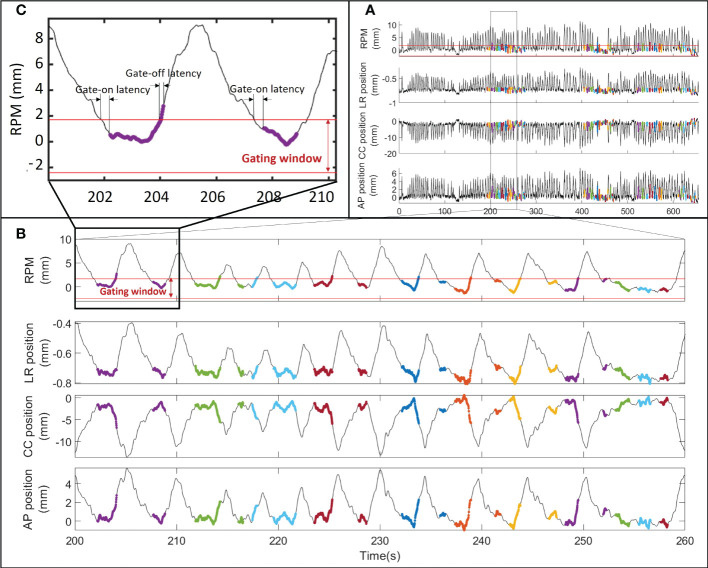
RPM signal and estimated internal tumor motion for fraction 1 of patient 3 during **(A)** the entire fraction, **(B)** a single field and **(C)** two breathing cycles. The thick colored curves show the spot delivery times with different energy layers indicated with different colors. The gating window and the gate-on and gate-off latencies are shown for the RPM signal.

**Figure 4** presents a typical example of the RPM signal and the ECM estimated tumor motion at a fraction, synchronized with the spot delivery times. Due to the gating latency the beam started 240 ms into the gating window and continued 80 ms after the RPM block moved out of the gating window (**Figure 4C**). The treatment error is reported in **Table 1** for each patient. Over all delivered spots the RMS treatment error was 1.3 mm (LR), 2.8 mm (CC), and 1.7 mm (AP), while the mean ( ± SD) 3D treatment error per patient was 3.9 mm ± 1.9 mm (patient 1), 3.7 mm ± 0.6 mm (patient 2) and 2.6 mm ± 1.7 mm (patient 3).

The maximum tumor motion range during a fraction was 6.4 mm (LR), 27.9 mm (CC), and 19.2 mm (AP) during field delivery independent of beam-on status and 2.7 mm (LR), 10 mm (CC), and 7.1 mm (AP) during beam-on periods. The mean tumor motion range during a single fraction was usually more than halved with gating compared to the full motion range (**Table 1**).

**Figure 5:**
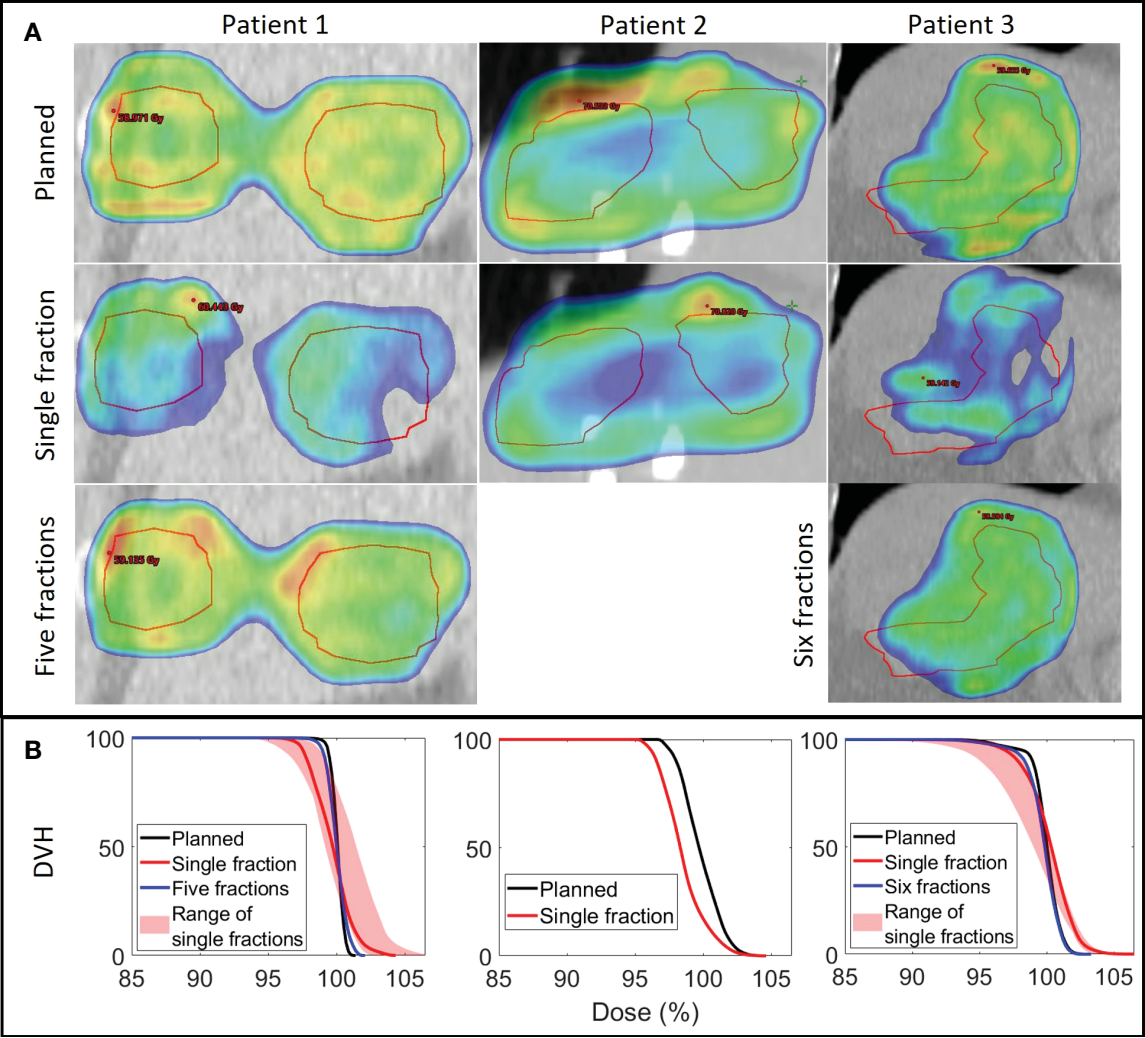
**(A)** Planned dose (1st row) and examples of reconstructed doses at a single fraction (2nd row) and averaged over all investigated fractions (3rd row) shown in a coronal plane through the center of the CTV (red contour) for each patient. Dose levels ≥ 95% are shown. Patient 2 only received one fraction. **(B)** Corresponding dose volume histograms (DVHs) for the CTV with the full range of single fraction DVHs indicated by the shaded area.

**Figure 6:**
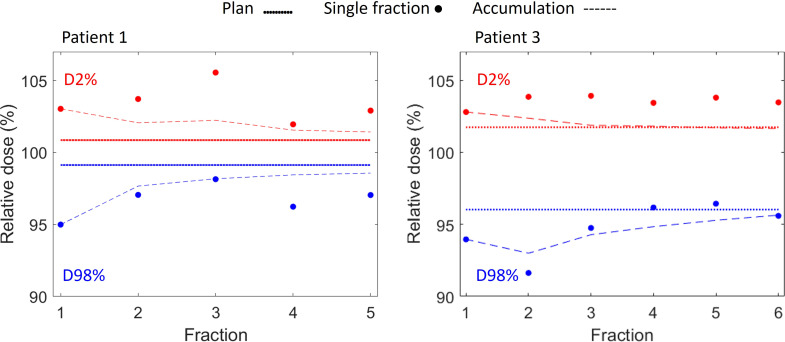
CTV D98% (blue) and D2% (red) for planned dose (solid lines), for reconstructed single fraction doses (dots) and for the cumulative reconstructed dose of 1-5 fractions (Patient 1, left) and 1-6 fractions (Patient 3, right) (dashed curves).

Large dose deterioration occurred at single fractions due to interplay effects with D2% being up to 4.7%-points higher than planned and D98% up to 4.4%-points lower than planned (**Figures 5**, **6**; **Table 2**). After 5-6 fractions the interplay effects tended to smear out due to averaging effects such that D2% and D98% converged towards the planned values (**Figure 6**). On average the CTV HI was larger than planned by 3.7%-points (range: 1.0-6.6%-points) for individual fractions and by 0.7%-points (range: 0.3-1.1%-points) for the average dose of 5 or 6 fractions (**Table 2**). The mean dose to the healthy liver tissue, averaged over all analyzed fractions, was different from the planned dose by 0.3%-points (patient 1), 1%-points (patient 2) and -0.1%-points (patient 3).

**Table 2:** D98%, D2% and homogeneity index (HI) for the CTV as planned and in the reconstructed doses for single fractions and averaged over all investigated fractions.

Patient	1			2			3		
Dose parameter	D98% (Gy)	D2% (Gy)	HI (%)	D98% (Gy)	D2% (Gy)	HI (%)	D98% (Gy)	D2% (Gy)	HI (%)
Planned	57.5	58.5	1.7	65.6	69.4	5.6	55.7	59.0	5.7
Single fraction (Mean ± SD)	56.1 ± 0.6	60.0 ± 0.7	6.7 ± 0.9	64.6	69.0	6.6	55.0 ± 0.9	60.0 ± 0.2	8.8 ± 1.7
5-6 fractions	57.2	58.8	2.9	–	–	–	55.4	58.9	6.0

Only one fraction was investigated for patient 2.

## 4 Discussion

With the present study we have developed and clinically demonstrated a method to estimate the internal target motion and its consequence on dose delivery during proton therapy of liver cancer at a standard equipped proton facility. As pointed out by recent international guidelines such monitoring is important for PBS proton therapy of moving targets but typically not commercially available (12, 13). The motion estimation was based on an ECM that was constructed at each treatment fraction using external RPM motion synchronized with internal 3D tumor motion extracted from CBCT projections. An in-house developed method that has been validated on a group of ten patients (11, 25) was subsequently applied to reconstruct the motion-including CTV dose. Considerable interplay effects at single fractions tended to smear out after more fractions.

Additionally, we investigated the accuracy of the online CBCT match to determine the exhale marker positions. Due to motion smearing and motion artifacts, the manual online match is subjective and prone to human errors, while the estimated marker trajectories provided an objective measure of the exhale position during CBCT. However, with differences between the online manual registration and offline trajectory-based marker match close to the resolution of the CT scan (2mm CC, ~1mm in-plane), the online match accuracy was acceptable. Yet, at a few individual fractions, slightly larger discrepancies in the CC direction were observed (up to 3.2 mm). Offline inspection of the online match revealed that these discrepancies could be ascribed to the online procedure being prone to human subjectivity and performed under time pressure with the patient waiting for treatment.

During CBCT scan and treatment delivery, large motion variations between individual respiratory cycles and total motion amplitudes of 2-3 cm, most prominent in the CC direction, were observed (**Figures 3**, **4**). Such motion is on par with previous studies of internal motion during radiotherapy of tumors in the liver (8, 32–35). Due to the extended time typically spent near the exhale phase of the respiratory cycle, limiting the treatment to an approximate 50% duty cycle around exhale more than halved the motion during beam-on and also reduced motion variation between treatment fractions (**Table 1**). The resulting mean geometrical treatment errors during proton spot delivery were thereby also limited to a few millimeters, including errors introduced by the rather small baseline shift of 0 - 3 mm typically observed between setup CBCTs and post-treatment CBCTs (**Table 1**). Notably, though not observed in the limited cohort of this study, baseline shifts and resulting treatment errors of higher magnitude can be expected during liver treatments of some patients (8, 33). Still, the initial findings of the present study confirmed the usability of externally guided respiratory gating, which has also been proposed by other groups for reducing the internal motion during liver proton therapy delivery (14–16).

Despite the use of respiraory gating, our dose reconstructions showed considereable dose deteriorations on a single-fraction level caused by interplay between proton spot delivery and target motion (**Figures 5**, **6**). This finding clinically confirms the simulation results of Zhang et al. who concluded that respiratory gating alone was insufficient to mitigate interplay effects in PBS proton therapy (16). However, fractionation tends to reduce interplay effects by averaging out local over and under dosage over several treatment fractions (36–39). In the present study, the interplay effects were almost averaged out after 5-6 fractions (**Figure 5**). Nevertheless, for hypofractionation treatments, one may need to combine gating with repainting (16, 40).

A few previous clinical studies investigated the dosimetric consequences of respiratory motion during particle therapy. Richter et al. (19) and Meijers et al. (21) combined machine log files with the spot delivery with an external respiratory signal obtained during treatment and used this to calculate the D4DD by distributing each spot delivery into corresponding phases of 4DCT scans. These studies also found that fractionation effectively mitigated interplay effects. For tumors in the thoraic region, anatomical changes such as presence of fluid or tumor shrinkage, caused more severe dosimetric changes (21). Limitations of the D4DD include neglectance of setup errors, the assumption of identical anatomy and respiratory motion amplitude at treatment as in the 4DCT and the dependence on deformable image registration for dose accumulation in a reference 4DCT phase. An advantage of the 4DCT based dose reconstruction, compared to our method, is that it includes estimations of dose degration caused by internal anatomical changes between individual 4DCT phases. Since our spot shift dose reconstruction was based on a single phase of the planning 4DCT it only considers the effects of rigid intrafraction motion. A potential improvement could be a hybrid dose reconstruction method that extends phase specific dose calculations in each 4DCT phase with spot shifts that accounts for the tumor motion during treatment that goes beyond the motion in the 4DCT scan. Such an extension of our method to individual 4DCT phases would improve the D4DD reconstruction method recommended by international guidelines to also include actual intrafraction motion and not only motion observed in 4DCTs (12, 13). However, a persisting challenge with such 4DCT dose accumulation is the reliance on deformable image registration and dose warping between CT scans, which is a procedure with considerable uncertainty (41).

In a recent and closely related study by Yamada et al. at Hokkaido University, machine log files were combined with intra-treatment monitoring of internal fiducial markers by stereoscopic x-ray fluoroscopy during liver proton therapy (24). The same dose reconstruction method as applied in the present study was used (25). Respiratory exhale gating guided by direct internal tumor motion monitoring was a big advantage of the study by Yamada et al. compared to ours. Tight internal gating windows of ±2 mm along each direction may also explain why Yamada et al. found considerably smaller mean 3D tumor position errors during spot delivery (0.8-1.3 mm) than the present study (2.6-3.9 mm) where only external monitoring with a gating window corresponding to approximately 50% duty cycle was applied. For comparison, a previous study on liver SBRT with internal exhale gating based on implanted electromagnetic markers and gating windows of ±3 mm (LR/AP) and ±4 mm (CC) found mean 3D tumor position errors of 1.2-3.0 mm (8). Despite the larger treatment errors in the present study the estimated delivered CTV dose was close to the planned dose when averaged over 5-6 fractions. Hence, the robust SFUD planning approach combined with CBCT-based setup to internal markers and fractionated exhale gated treatments provided appropriate mitigation of intrafraction motion. Further studies including more patients and the effects of inter-fraction deformations (e.g., by dose reconstruction on weekly control 4DCT scans) are necessary to conclude on the overall treatment quality. For example, deformations may affect the proton range and the assumption that implanted markers serve as an accurate surrogate for the CTV position, especially if they are not implanted near the tumor (42). Ultimately, comprehensive all-inclusive fraction-specific dose reconstruction and dose accumulation could be used to trigger plan-adaptations in case of unacceptable dose coverage.

A limitation of the current study is the indirect estimation of the tumor motion during treatment by an ECM. Although an ECM of the day was built, the accuracy cannot be expected to be better than the ECM fit, which had mean RMS errors of almost half of the treatment errors and exceeding 2 mm in the CC direction for one patient (**Table 1**). Furthermore, high intrafraction stability of the ECM with the ability to detect internal baseline shift cannot in general be assumed (43–46). Optimally, the ECM should be built based on the setup CBCT and then validated by the post-treatment CBCT. However, phantom tests showed discrepancies between couch shifts and changes in the RPM signal that hindered correct adjustment of an ECM from a setup CBCT to allow usage after setup couch corrections. For this reason, intra-treatment motion could only be investigated for fractions with a post-treatment CBCT in this study. The ECM stability issue may be addressed by capturing a series of stereoscopic images before each field delivery to confirm and update the ECM.

It is worth noting that the RPM system available for proton therapy clearly lags behind the optical monitoring system available from the same vendor for photon radiotherapy (TrueBeam, Varian) with a stereoscopic camera that allows adaptation of the ECM to couch shifts (47). Furthermore, the cumbersome manual synchronizations of the RPM log files with CBCT projections and treatment machine log files in the current study (**Figure 1**) would not be needed if the respiratory monitoring, imaging, and beam delivery systems were similarly well-integrated as on a TrueBeam linear accelerator. The manual synchronization of RPM log files with machine log files was only possible in this study because the beam pauses in the machine log files had a unique temporal pattern that could be matched with the RPM gating signal (**Figure 1E**). This synchronization would not be possible for non-gated treatments. Since the synchronization can only be performed post-treatment, it is currently a barrier for online real-time dose reconstruction, which has been demonstrated clinically for liver SBRT (48), but could be even more relevant for proton PBS.

In summary, dose reconstruction including the effects of setup errors, rigid motion, interplay effects and the smearing hereof after 5-6 fractions was performed for HCC proton therapy. For the included patients, it showed that our treatment strategy of exhale gating resulted in an acceptable CTV dose coverage. With a smoother workflow and automation this could be used to trigger a plan adaptation if the CTV dose coverage turned out to be unacceptable. Since CTV dose deficits could also be caused by interfractional changes, the motion-including dose reconstruction should ideally be extended to account for such changes, for example by applying it on the anatomy of weekly 4DCTs.

## 5 Conclusion

A method to estimate internal tumor motion and reconstruct the motion-including fraction dose for PBS proton therapy in the liver was developed and successfully demonstrated clinically at a conventional proton facility.

## Data availability statement

The original contributions presented in the study are included in the article/supplementary material. Further inquiries can be directed to the corresponding author.

## Ethics statement

The studies involving human participants were reviewed and approved by the regional research ethics committee and the regional register of research projects (ref. number 742503, case number 1-16-02-320-21). The research was conducted in accordance with the principles of the Helsinki Declaration and local statutory requirements. The patients/participants provided their written informed consent to participate in this study.

## Author contributions

EW, HM, BW, MH, JT, LS, PP designed the treatment and imaging protocol for the study. PP, SN, EW conceived, designed and drafted the manuscript and all authors revised the manuscript. JT, LS, SN, EW collected the data. Software was developed by JB (marker segmentation) and PP (segmentation correction, 3D trajectory estimation, data synchronization, dose reconstruction), and streamlined by SN who performed the data analysis. All authors contributed to the article and approved the submitted version.

## References

[1] Benson AB D’Angelica MI Abbott DE Abrams TA Alberts SR Anaya DA . Nccn guidelines insights: Hepatobiliary cancers, version 1.2017. J Natl Compr Cancer Network (2017) 15(5):563–73. doi: 10.6004/jnccn.2017.0059 PMC5557008 28476736

[2] Cheng JC-H Wu J-K Lee PC-T Liu H-S Jian JJ-M Lin Y-M . Biologic susceptibility of hepatocellular carcinoma patients treated with radiotherapy to radiation-induced liver disease. Int J Radiat Oncol Biol Phys (2004) 60(5):1502–9. doi: 10.1016/j.ijrobp.2004.05.048 15590181

[3] Miften M Vinogradskiy Y Moiseenko V Grimm J Yorke E Jackson A . Radiation dose-volume effects for liver sbrt. Int J Radiat Oncol Biol Phys (2021) 110(1):196–205. doi: 10.1016/j.ijrobp.2017.12.290 29482870 PMC6095822

[4] Lomax AJ Pedroni E Rutz HP Goitein G . The clinical potential of intensity modulated proton therapy. Z für Medizinische Physik (2004) 14(3):147–52. doi: 10.1078/0939-3889-00217 15462415

[5] Yoo GS Yu JI Park HC . Proton therapy for hepatocellular carcinoma: Current knowledges and future perspectives. World J Gastroenterol (2018) 24(28):3090. doi: 10.3748/wjg.v24.i28.3090 30065555 PMC6064962

[6] Kobeissi JM Hilal L Simone CB 2nd Lin H Crane CH Hajj C . Proton therapy in the management of hepatocellular carcinoma. Cancers (2022) 14(12):2900. doi: 10.3390/cancers14122900 35740567 PMC9220794

[7] Spychalski P Kobiela J Antoszewska M Błażyńska-Spychalska A Jereczek-Fossa BA Høyer M . Patient specific outcomes of charged particle therapy for hepatocellular carcinoma–a systematic review and quantitative analysis. Radiotherapy Oncol (2019) 132:127–34. doi: 10.1016/j.radonc.2018.12.012 30825961

[8] Worm ES Høyer M Hansen R Larsen LP Weber B Grau C . A prospective cohort study of gated stereotactic liver radiation therapy using continuous internal electromagnetic motion monitoring. Int J Radiat Oncol Biol Phys (2018) 101(2):366–75. doi: 10.1016/j.ijrobp.2018.02.010 29559289

[9] Kooy H Grassberger C . Intensity modulated proton therapy. Br J Radiol (2015) 88(1051):20150195. doi: 10.1259/bjr.20150195 26084352 PMC4628542

[10] Rietzel E Bert C . Respiratory motion management in particle therapy. Med Phys (2010) 37(2):449–60. doi: 10.1118/1.3250856 20229853

[11] Worm ES Hansen R Høyer M Weber B Mortensen H Poulsen PR . Uniform versus non-uniform dose prescription for proton stereotactic body radiotherapy of liver tumors investigated by extensive motion-including treatment simulations. Phys Med Biol (2021) 66(20):205009. doi: 10.1088/1361-6560/ac2880 34544071

[12] Li H Dong L Bert C Chang J Flampouri S Jee KW . AAPM task group report 290: Respiratory motion management for particle therapy. Med Phys (2022) 49(4):e50–81. doi: 10.1002/mp.15470 PMC9306777 35066871

[13] Chang JY Zhang X Knopf A Li H Mori S Dong L . Consensus guidelines for implementing pencil-beam scanning proton therapy for thoracic malignancies on behalf of the ptcog thoracic and lymphoma subcommittee. Int J Radiat Oncol Biol Phys (2017) 99(1):41–50. doi: 10.1016/j.ijrobp.2017.05.014 28816159

[14] Lu HM Brett R Sharp G Safai S Jiang S Flanz J . A respiratory-gated treatment system for proton therapy. Med Phys (2007) 34(8):3273–8. doi: 10.1118/1.2756602 17879790

[15] Gelover E Deisher AJ Herman MG Johnson JE Kruse JJ Tryggestad EJ . Clinical implementation of respiratory-gated spot-scanning proton therapy: An efficiency analysis of active motion management. J Appl Clin Med Phys (2019) 20(5):99–108. doi: 10.1002/acm2.12584 PMC6523004 30972922

[16] Zhang Y Huth I Weber DC Lomax AJ . A statistical comparison of motion mitigation performances and robustness of various pencil beam scanned proton systems for liver tumour treatments. Radiotherapy Oncol (2018) 128(1):182–8. doi: 10.1016/j.radonc.2018.01.019 29459153

[17] Mizuhata M Takamatsu S Shibata S Bou S Sato Y Kawamura M . Respiratory-gated proton beam therapy for hepatocellular carcinoma adjacent to the gastrointestinal tract without fiducial markers. Cancers (2018) 10(2):58. doi: 10.3390/cancers10020058 29466294 PMC5836090

[18] Sharp GC Lu HM Trofimov A Tang X Jiang SB Turcotte J . Assessing residual motion for gated proton-beam radiotherapy. J Radiat Res (2007) 48(Suppl_A):A55–A9. doi: 10.1269/jrr.48.A55 17513900

[19] Richter D Saito N Chaudhri N Härtig M Ellerbrock M Jäkel O . Four-dimensional patient dose reconstruction for scanned ion beam therapy of moving liver tumors. Int J Radiat Oncol Biol Phys (2014) 89(1):175–81. doi: 10.1016/j.ijrobp.2014.01.043 24725700

[20] Meijers A Jakobi A Stützer K Guterres Marmitt G Both S Langendijk J . Log file-based dose reconstruction and accumulation for 4D adaptive pencil beam scanned proton therapy in a clinical treatment planning system: Implementation and proof-of-Concept. Med Phys (2019) 46(3):1140–9. doi: 10.1002/mp.13371 30609061

[21] Meijers A Knopf A-C Crijns AP Ubbels JF Niezink AG Langendijk JA . Evaluation of interplay and organ motion effects by means of 4D dose reconstruction and accumulation. Radiotherapy Oncol (2020) 150:268–74. doi: 10.1016/j.radonc.2020.07.055 32768509

[22] Worm ES Høyer M Fledelius W Hansen AT Poulsen PR . Variations in magnitude and directionality of respiratory target motion throughout full treatment courses of stereotactic body radiotherapy for tumors in the liver. Acta Oncol (2013) 52(7):1437–44. doi: 10.3109/0284186X.2013.813638 23879645

[23] Rankine L Wan H Parikh P Maughan N Poulsen P DeWees T . Cone-beam computed tomography internal motion tracking should be used to validate 4-dimensional computed tomography for abdominal radiation therapy patients. Int J Radiat Oncol Biol Phys (2016) 95(2):818–26. doi: 10.1016/j.ijrobp.2016.01.047 27020102

[24] Yamada T Takao S Koyano H Nihongi H Fujii Y Hirayama S . Validation of dose distribution for liver tumors treated with real-Time-Image gated spot-scanning proton therapy by log data based dose reconstruction. J Radiat Res (2021) 62(4):626–33. doi: 10.1093/jrr/rrab024 PMC8273791 33948661

[25] Colvill E Petersen JB Hansen R Worm E Skouboe S Høyer M . Validation of fast motion-including dose reconstruction for proton scanning therapy in the liver. Phys Med Biol (2018) 63(22):225021. doi: 10.1088/1361-6560/aaeae9 30457119

[26] Bertholet J Wan H Toftegaard J Schmidt M Chotard F Parikh P . Fully automatic segmentation of arbitrarily shaped fiducial markers in cone-beam CT projections. Phys Med Biol (2017) 62(4):1327. doi: 10.1088/1361-6560/aa52f7 28114115

[27] Poulsen PR Cho B Keall PJ . A method to estimate mean position, motion magnitude, motion correlation, and trajectory of a tumor from cone-beam CT projections for image-guided radiotherapy. Int J Radiat Oncol Biol Phys (2008) 72(5):1587–96. doi: 10.1016/j.ijrobp.2008.07.037 19028282

[28] Worm ES Høyer M Fledelius W Nielsen JE Larsen LP Poulsen PR . On-line use of three-dimensional marker trajectory estimation from cone-beam computed tomography projections for precise setup in radiotherapy for targets with respiratory motion. Int J Radiat Oncol Biol Phys (2012) 83(1):e145–51. doi: 10.1016/j.ijrobp.2011.12.007 22516384

[29] Worm E Thomsen J Johansen J Poulsen P . Oc-0040 gating latencies and resulting geometrical errors at clinical proton and photon accelerators. Radiotherapy Oncol (2022) 170:S13–S5. doi: 10.1016/S0167-8140(22)02459-8

[30] Poulsen PR Eley J Langner U Simone CB II Langen K . Efficient interplay effect mitigation for proton pencil beam scanning by spot-adapted layered repainting evenly spread out over the full breathing cycle. Int J Radiat Oncol Biol Phys (2018) 100(1):226–34. doi: 10.1016/j.ijrobp.2017.09.043 29254775

[31] Ruan D Fessler JA Balter JM Berbeco R Nishioka S Shirato H . Inference of hysteretic respiratory tumor motion from external surrogates: A state augmentation approach. Phys Med Biol (2008) 53(11):2923. doi: 10.1088/0031-9155/53/11/011 18460744

[32] Park JC Park SH Kim JH Yoon SM Song SY Liu Z . Liver motion during cone beam computed tomography guided stereotactic body radiation therapy. Med Phys (2012) 39(10):6431–42. doi: 10.1118/1.4754658 23039678

[33] Worm ES Høyer M Fledelius W Poulsen PR . Three-dimensional, time-resolved, intrafraction motion monitoring throughout stereotactic liver radiation therapy on a conventional linear accelerator. Int J Radiat Oncol Biol Phys (2013) 86(1):190–7. doi: 10.1016/j.ijrobp.2012.12.017 23414764

[34] Poulsen PR Worm ES Petersen JB Grau C Fledelius W Høyer M . Kilovoltage intrafraction motion monitoring and target dose reconstruction for stereotactic volumetric modulated arc therapy of tumors in the liver. Radiotherapy Oncol (2014) 111(3):424–30. doi: 10.1016/j.radonc.2014.05.007 24997991

[35] Xu Q Hanna G Grimm J Kubicek G Pahlajani N Asbell S . Quantifying rigid and nonrigid motion of liver tumors during stereotactic body radiation therapy. Int J Radiat Oncol Biol Phys (2014) 90(1):94–101. doi: 10.1016/j.ijrobp.2014.05.007 25195990

[36] Dolde K Zhang Y Chaudhri N Dávid C Kachelrieß M Lomax AJ . 4DMRI-based investigation on the interplay effect for pencil beam scanning proton therapy of pancreatic cancer patients. Radiat Oncol (2019) 14(1):1–13. doi: 10.1186/s13014-019-1231-2 30732657 PMC6367829

[37] Grassberger C Dowdell S Lomax A Sharp G Shackleford J Choi N . Motion interplay as a function of patient parameters and spot size in spot scanning proton therapy for lung cancer. Int J Radiat Oncol Biol Phys (2013) 86(2):380–6. doi: 10.1016/j.ijrobp.2013.01.024 PMC3646997 23462423

[38] Dowdell S Grassberger C Sharp G Paganetti H . Interplay effects in proton scanning for lung: A 4D Monte Carlo study assessing the impact of tumor and beam delivery parameters. Phys Med Biol (2013) 58(12):4137. doi: 10.1088/0031-9155/58/12/4137 23689035 PMC3752993

[39] Li H Li Y Zhang X Li X Liu W Gillin MT . Dynamically accumulated dose and 4D accumulated dose for moving tumors. Med Phys (2012) 39(12):7359–67. doi: 10.1118/1.4766434 PMC3523466 23231285

[40] Furukawa T Inaniwa T Sato S Shirai T Mori S Takeshita E . Moving target irradiation with fast rescanning and gating in particle therapy. Med Phys (2010) 37(9):4874–9. doi: 10.1118/1.3481512 20964205

[41] Ribeiro CO Knopf A Langendijk JA Weber DC Lomax AJ Zhang Y . Assessment of dosimetric errors induced by deformable image registration methods in 4D pencil beam scanned proton treatment planning for liver tumours. Radiotherapy Oncol (2018) 128(1):174–81. doi: 10.1016/j.radonc.2018.03.001 29571904

[42] Wunderink W Romero AM Seppenwoolde Y De Boer H Levendag P Heijmen B . Potentials and limitations of guiding liver stereotactic body radiation therapy set-up on liver-implanted fiducial markers. Int J Radiat Oncol Biol Phys (2010) 77(5):1573–83. doi: 10.1016/j.ijrobp.2009.10.040 20399034

[43] Ge J Santanam L Yang D Parikh PJ . Accuracy and consistency of respiratory gating in abdominal cancer patients. Int J Radiat Oncol Biol Phys (2013) 85(3):854–61. doi: 10.1016/j.ijrobp.2012.05.006 22717241

[44] Pettersson N Oderinde OM Murphy J Simpson D Cerviño LI . Intrafractional relationship changes between an external breathing signal and fiducial marker positions in pancreatic cancer patients. J Appl Clin Med Phys (2020) 21(3):153–61. doi: 10.1002/acm2.12841 PMC7075406 32170900

[45] Takao S Miyamoto N Matsuura T Onimaru R Katoh N Inoue T . Intrafractional baseline shift or drift of lung tumor motion during gated radiation therapy with a real-time tumor-tracking system. Int J Radiat Oncol Biol Phys (2016) 94(1):172–80. doi: 10.1016/j.ijrobp.2015.09.024 26700711

[46] Ren Q Nishioka S Shirato H Berbeco R . Adaptive external gating based on the updating method of Internal/External correlation and gating window before each beam delivery. Phys Med Biol (2012) 57(9):N145. doi: 10.1088/0031-9155/57/9/N145 22507921

[47] Bertholet J Toftegaard J Hansen R Worm ES Wan H Parikh PJ . Automatic online and real-time tumour motion monitoring during stereotactic liver treatments on a conventional linac by combined optical and sparse monoscopic imaging with kilovoltage X-rays (Cosmik). Phys Med Biol (2018) 63(5):055012. doi: 10.1088/1361-6560/aaae8b 29516868

[48] Skouboe S Ravkilde T Bertholet J Hansen R Worm ES Muurholm CG . First clinical real-time motion-including tumor dose reconstruction during radiotherapy delivery. Radiotherapy Oncol (2019) 139:66–71. doi: 10.1016/j.radonc.2019.07.007 31431367

